# Thyroid hormone status defines brown adipose tissue activity and browning of white adipose tissues in mice

**DOI:** 10.1038/srep38124

**Published:** 2016-12-12

**Authors:** Juliane Weiner, Mathias Kranz, Nora Klöting, Anne Kunath, Karen Steinhoff, Eddy Rijntjes, Josef Köhrle, Vilia Zeisig, Mohammed Hankir, Claudia Gebhardt, Winnie Deuther-Conrad, John T. Heiker, Susan Kralisch, Michael Stumvoll, Matthias Blüher, Osama Sabri, Swen Hesse, Peter Brust, Anke Tönjes, Kerstin Krause

**Affiliations:** 1 Department of Endocrinology and Nephrology, University Hospital, Leipzig, Germany; 2 Helmholtz-Zentrum Dresden-Rossendorf, Institute of Radiopharmaceutical Cancer Research, Research Site Leipzig, Department of Neuroradiopharmaceuticals, Leipzig, Germany; 3 University of Leipzig, IFB Adiposity Diseases, Leipzig, Germany; 4 German Center for Diabetes Research (DZD), Leipzig, Germany; 5 Department of Nuclear Medicine, University Hospital, Leipzig, Germany; 6 Institute of Experimental Endocrinology, Charité University Hospital, Berlin, Germany

## Abstract

The present study aimed to determine the effect of thyroid hormone dysfunction on brown adipose tissue activity and white adipose tissue browning in mice. Twenty randomized female C57BL/6NTac mice per treatment group housed at room temperature were rendered hypothyroid or hyperthyroid. *In-vivo* small animal ^18^F-FDG PET/MRI was performed to determine the effects of hypo- and hyperthyroidism on BAT mass and BAT activity. *Ex-vivo*^14^C-acetate loading assay and assessment of thermogenic gene and protein expression permitted analysis of oxidative and thermogenic capacities of WAT and BAT of eu-, hyper and hypothyroid mice. ^18^F-FDG PET/MRI revealed a lack of brown adipose tissue activity in hypothyroid mice, whereas hyperthyroid mice displayed increased BAT mass alongside enhanced ^18^F-FDG uptake. In white adipose tissue of both, hyper- and hypothyroid mice, we found a significant induction of thermogenic genes together with multilocular adipocytes expressing UCP1. Taken together, these results suggest that both the hyperthyroid and hypothyroid state stimulate WAT thermogenesis most likely as a consequence of enhanced adrenergic signaling or compensation for impaired BAT function, respectively.

Thyroid hormones (TH) are intimately involved in the regulation of energy metabolism. Administration of TH to mammals leads to an increase in basal metabolic rate and thermogenesis^1^. The important role of TH in the regulation of body temperature homeostasis is perhaps best reflected by the cold or heat intolerance of animals and humans with hypothyroidism or hyperthyroidism, respectively^2^. Under conditions of thermoneutrality, core body temperature can be maintained in the absence of any thermoregulatory process (e.g. shivering). However, under conditions of cold exposure adaptive thermogenesis occurs through a compensatory increase in sympathetic nervous system tone (SNS) which, in concert with TH, accelerates energy expenditure (EE) and increases body temperature^3^.

Numerous studies primarily performed on animal models have investigated the mechanism of TH for activating brown adipose tissue (BAT). Findings from different TR knockout mice (e.g. TRα, TRβ, TRα1+m) and the use of isoform-selective agonists indicate specific roles for TH-adrenergic interactions in BAT and white adipose tissue(WAT)^4^,^5^,^6^,^7^,^8^,^9^,^10^. Early studies in thyroidectomized and hypothyroid rats demonstrated the relevance of BAT specific type II iodothyronine deiodinase Dio2 as a local and systemic source of 3,3′,5-triiodothyronine (T_3_) under cold exposure and for the first time provided evidence for T_3_ in the enhancement of the thermogenic response to sympathetic stimulation^11^,^12^. This suggests that the subsequent heat liberation is actually a result of synergism between norepinephrine (NE) and T_3_ signaling^13^. The significance of the central nervous system in TH activation of BAT was demonstrated in rats in which central administration of T_3_, via the inhibition of hypothalamic AMP-activated protein kinase (AMPK), induces thermogenic gene expression in BAT through stimulation of sympathetic nervous system (SNS) activity^14^. Additionally, SNS stimulation results in activation of β-adrenergic receptors (β-AR) by NE which induces both the enhanced thermogenic activity of existing brown adipocytes and the recruitment of new cells to BAT depots^1^. Energy release is accomplished by the activity of UCP1, a BAT-specific mitochondrial uncoupling protein^1^. With regards to WAT, a distinct population of UCP1-positive adipocytes arises within subcutaneous and visceral depots of mice in response to cold exposure^15^,^16^ and β_3_ adrenergic receptor agonist treatment^15^,^17^,^18^. To date it remains a matter of controversy as to whether these cells, entitled “brite” or “beige” adipocytes, are produced from the conversion of existing white adipocytes and/or the differentiation of WAT resident progenitor cells, leading to a process called “browning”^19^,^20^,^21^.

There is preliminary evidence that suggests a role for TH in the induction of beige adipocytes in WAT. In human multipotent adipose-derived stem cells, 3–10 day treatment with T_3_ induced UCP1 expression which was most pronounced during the differentiation phase and was dependent on the presence of thyroid hormone receptor β (TRβ)^22^. Similarly, treatment of mice with the TRβ specific agonist GC-1 leads to induction of adaptive thermogenesis in subcutaneous WAT, increased energy expenditure, and restoration of cold tolerance in cold-intolerant mice independent from BAT activity^23^,^24^. Furthermore, in mice centrally administered T3 leads to the recruitment of BAT in inguinal WAT which is accompanied by increased energy expenditure and body temperature. Since the effect was abolished in UCP1 KO mice, the findings highlight the significance of UCP1 as a central relais modulating the central effects of TH^25^. Furthermore, a novel circuit for the control of TH feedback in the brain via the Liver X receptor α and β has recently been published^26^. LXRαβ^−/−^ mice display increased TH serum level combined with changes in expression of genes associated with TH synthesis and TH transport as well as prominent UCP1 staining in the subcutaneous adipose depot^26^. In a positron emission tomography (PET) study of a patient with a history of thyroid cancer, systemic administration of TH led to BAT activation in periumbilical WAT indicating that browning had occurred as a result of T_4_ supplementation for 14 days^27^.

Despite the significance of TH for adaptive thermogenesis, there is currently limited understanding of whether and how different thyroid states, i.e. hypo- and hyperthyroidism, regulate BAT thermogenesis as well as the thermogenic activity of WAT. Therefore, the present study aimed to determine TH induced effects on (I) BAT activity and (II) WAT browning in a mouse model of thyroidal dysfunction with a view to incorporate the results into the context of TH-regulated effects on whole-body energy homeostasis.

## Results

### Both hypo- and hyperthyroidism induce brown fat biogenesis

**Figure 1:**
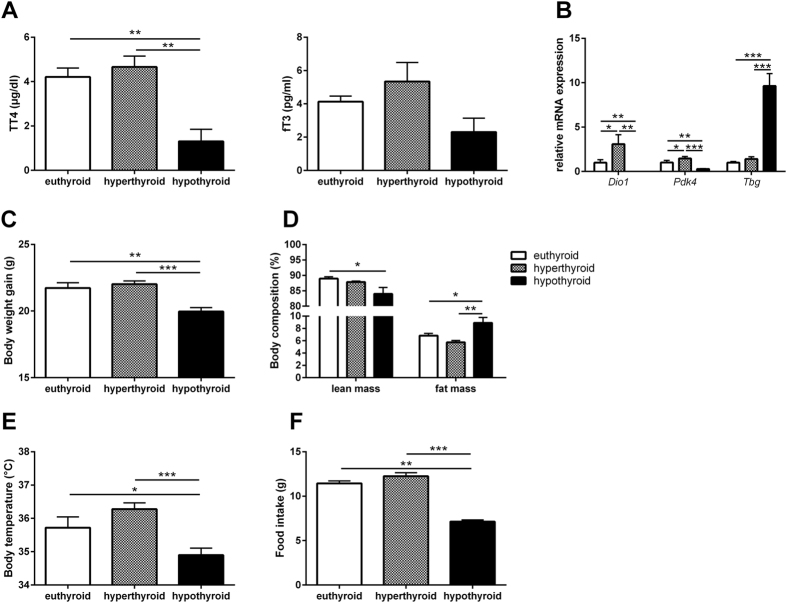
Thyroid state and phenotypic characterization of hyper- hypo- and euthyroid mice. (**A**) Total T4 and free T3 serum concentrations, (**B**) Hepatic expression of TH-responsive genes, (**C**) Final body weight and (**D**) Lean and fat mass and (**E**) Body temperature and (**F**) food intake of eu-, hyper-, and hypothyroid mice. n = 10 mice per group; data are presented as mean and SEM (*p < 0.05, **p < 0.01, ***p < 0.001).

Comparison of total T_4_ and free T_3_ serum concentrations between eu-, hyper- and hypothyroid mice confirmed the hyperthyroid and hypothyroid state of the animals (Fig. 1A). This finding is further substantiated by the differential hepatic expression of the TH-responsive genes deiodinase 1 (*Dio1*), thyroxine-binding globulin (*Tbg*) and pyruvate dehydrogenase kinase 4 (*Pdk4*) (Fig. 1B). The mRNA level of *Dio1* and *Pdk4* in liver increased significantly upon T_4_-treatment and decreased significantly in hypothyroid animals. In addition, induction of *Tbg*, negatively regulated by TH, confirmed the hypothyroid state (Fig. 1B).

Hyperthyroid mice showed slightly enhanced weight gain at the end of treatment while hypothyroid animals displayed significantly decreased body weights (Fig. 1C). While there was no change in body composition between hyperthyroid and euthyroid animals, hypothyroid mice had significantly reduced lean mass and concurrently increased body fat (8.9 ± 0.9%) compared with euthyroid (6.8 ± 0.3%) and hyperthyroid mice (5.7 ± 0.3%; Fig. 1D). Core body temperature was significantly decreased in hypothyroid mice (34.9 ± 0.2 °C) compared with hyperthyroid (36.2 ± 0.2 °C) and euthyroid mice (35.7 ± 0.3 °C); Fig. 1E). Furthermore, food intake decreased in hypothyroid animals (Fig. 1F).

**Figure 2:**
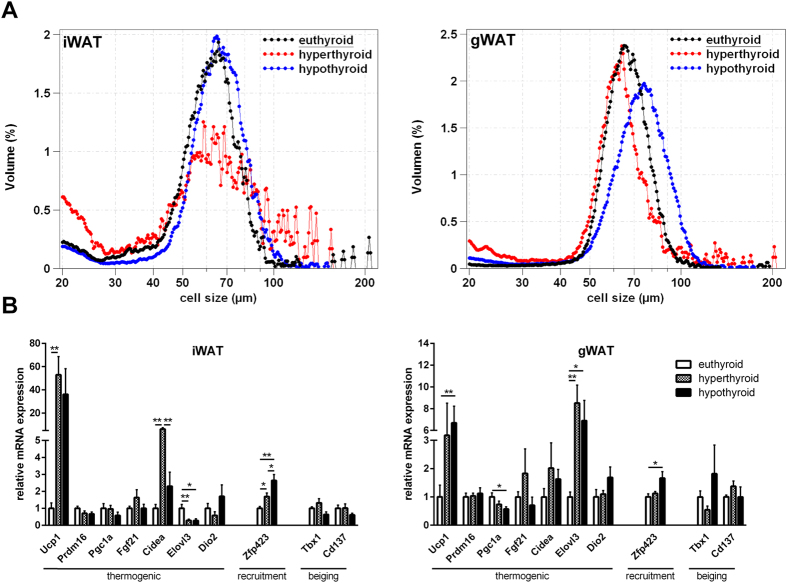
Histology and gene thermogenic expression in WAT. (**A**) Cell size distribution of adipocytes from gWAT and iWAT (n = 10/group); (**B**) Expression of genes associated with thermogenesis, recruitment, beiging and β-adrenergic signaling (n = 7–10/group). Data are presented as mean and SEM (*p < 0.05, **p < 0.01, ***p < 0.001).

In view of the striking differences in core body temperature and body fat distribution between hyper- and hypothyroid mice, we next asked to which extent WAT depot contributes to TH induced thermogenesis. Consistent with the finding of increased fat mass, adipocytes from hypothyroid mice were larger in size with increased lipid accumulation in iWAT and gWAT when compared with hyper- and euthyroid mice (Fig. 2A). In addition we observed moderate increases in thermogenic gene program in the white fat depots of both hypo- and hyperthyroid mice as compared to euthyroid littermates. Strikingly, *Ucp1* gene expression was upregulated by about 40-fold in iWAT of hypo- and hyperthyroid mice compared to euthyroid controls. Moreover, we observed a significant increase in *Ucp1* mRNA in gWAT, which is considered to have a much lower capacity to induce thermogenic gene programs (Fig. 2B). Of interest, the zinc finger protein *Zfp423*, which is an essential determinant of preadipocyte commitment^28^,^29^, was predominantly upregulated in WAT of hypothyroid mice (Fig. 2B).

**Figure 3:**
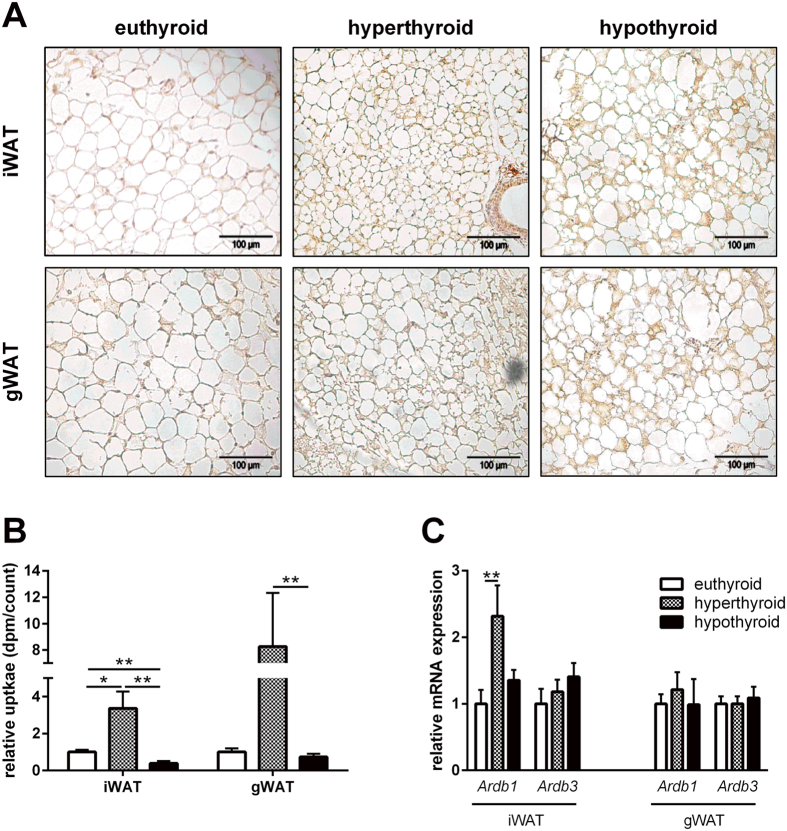
UCP-1 expression and fatty acid oxidation in WAT. (**A**) Representative Ucp1 immunohistochemical stainings in gWAT and iWAT in eu-, hyper- and hypothyroid mice; (**B**) *ex-vivo* 14C-palmitate uptake in n = 10 mice/group. (**C**) *Ardb1* and *Ardb3* mRNA expression in gWAT and iWAT (n = 5 mice/group). Data are presented as mean and SEM (*p < 0.05; **p < 0.01).

Histological examination of the white fat depots revealed the presence of abundant islets with a distinctive multilocular appearance of adipocytes which were positively stained for UCP1 in both, hyper- and hypothyroid states (Fig. 3A).

We then investigated whether the presence of brown fat-like cells has consequences for fatty acid oxidation. As demonstrated in Fig. 3B, the highest uptake of ^14^C-acetate was found in both iWAT and gWAT of hyperthyroid mice in comparison with hypo- and euthyroid mice. This was accompanied by a significantly higher level of *Ardb1* expression in iWAT of hyperthyroid mice, whereas the expression *Ardb1* and *Ardb3* in gWAT of hypo- and euthyroid mice did not change (Fig. 3C).

### Hypothyroidism is associated with enhanced β_3_-adrenergic tone in BAT

**Figure 4:**
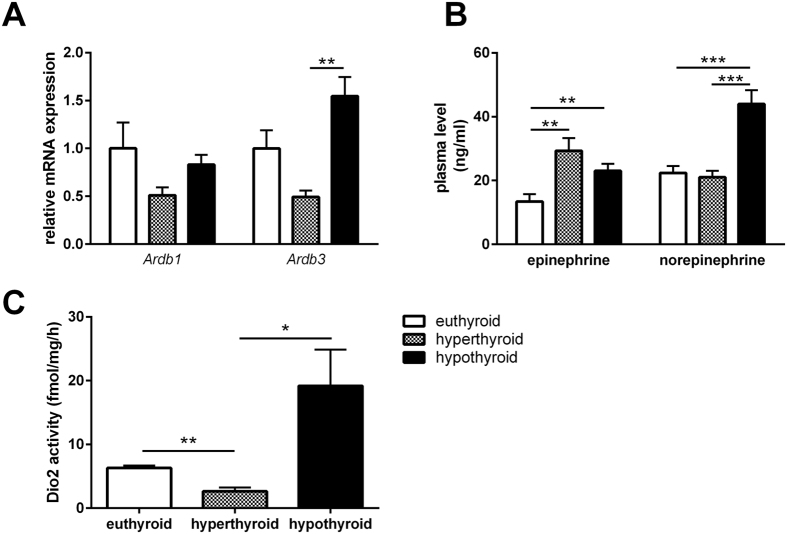
Characterization of the thyroid-adrenergic axis. (**A**) Expression of *Ardb1* and *Ardb3* in BAT (n = 7–10/group). (**B**) Circulating levels of epinephrine and norepinephrine (n = 10/group); (**C**) BAT DIO2 activity (n = 4–5/group). Data are presented as mean and SEM (*p < 0.05, **p < 0.01, ***p < 0.001).

Given the differences in core body temperature despite the activation of the thermogenic program in WAT at room temperature, we next asked whether this is due to changes in the thyroid-adrenergic axis induced by thyroidal dysfunction. Immunohistochemical staining of tyrosine hydroxylase, which is the rate-limiting enzyme for catecholamine synthesis^30^, revealed no differences in abundance in the BAT of hyperthyroid and hypothyroid mice (data not shown). Furthermore, while we did not observe changes in gene expression of the β-adrenergic receptor 1 (*Ardb1*) in BAT, there was a significant increase in gene expression of *Ardb3* in BAT of hypo- vs hyperthyroid mice (Fig. 4A).

BAT is profusely innervated by sympathetic nerve terminals with norepinephrine (NE) acting via β-ARs^31^. Therefore, we next determined the concentrations of circulating NE and epinephrine in the experimental groups. Intriguingly, we found that concentrations of NE only increased in hypothyroid mice whereas epinephrine increased in both hyperthyroid and hypothyroid mice compared to euthyroid mice (Fig. 4B). The activity of the Dio2 in hypothyroid BAT was 20-fold increased compared to hyperthyroid mice (p < 0.05). In hyperthyroid BAT Dio2 activity was significantly lower than in euthyroid controls (p < 0.01; Fig. 4C).

### Different morphology and gene expression in BAT of hypo- and hyperthyroid mice

In order to further unravel the discrepancies between hypothermia despite increased adrenergic signalling and WAT browning in hypothyroid mice we next investigated BAT metabolism.

**Figure 5:**
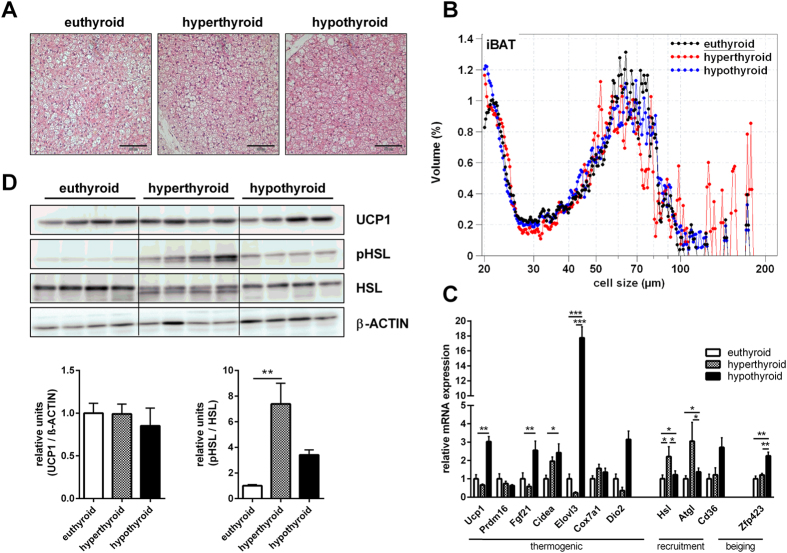
Characterization of BAT. (**A**) Representative hematoxylin and eosin sections; (**B**) Lipid droplet size and number (n = 10/group); (**C**) Expression of genes associated with thermogenesis, lipolysis, recruitment and β-adrenergic signaling (n = 7–10/group); (**D**) Quantification of BAT UCP1, pHSL and HSL protein by Western Blot analysis and normalization against β-actin as loading control (n = 4 mice/group). Data are presented as mean and SEM (*p < 0.05, **p < 0.01, ***p < 0.001).

Histological examination of interscapular BAT revealed gross differences in cell morphology. Whereas BAT of euthyroid mice contained mixed regions of white and brown adipocytes, the BAT of hypothyroid mice contained predominantly adipocytes with unilocular lipid droplets of intermediate size between WAT and BAT. Hyperthyroid BAT displayed a distinct morphology with a decreased cell size of the mainly multilocular adipocytes (Fig. 5A+B).

Gene expression analysis of thermogenic markers, including *Ucp1*, *Fgf21, Cidea, Dio2 and Elovl3,* revealed a remarkable collective overexpression in the hypothyroid BAT compared with hyperthyroid mice (Fig. 5C). However, the high induction of *Ucp1* mRNA in hypothyroid mice was not reflected on the level of UCP1 protein expression. Furthermore, there was no difference in UCP1 protein expression between hyperthyroid and euthyroid mice (Fig. 5D). In contrast, in hyperthyroid BAT we found an increased activation of β-adrenergic signaling as demonstrated by higher gene expression of the hormone-sensitive lipase (*Hsl*) and adipose triglyceride lipase (*Atgl;* Fig. 5C) together with an increased Ser660-phosphorylation of HSL (Fig. 5D).

### Volume and activity of BAT is distinctively regulated in hypo- and hyperthyroid mice

**Figure 6:**
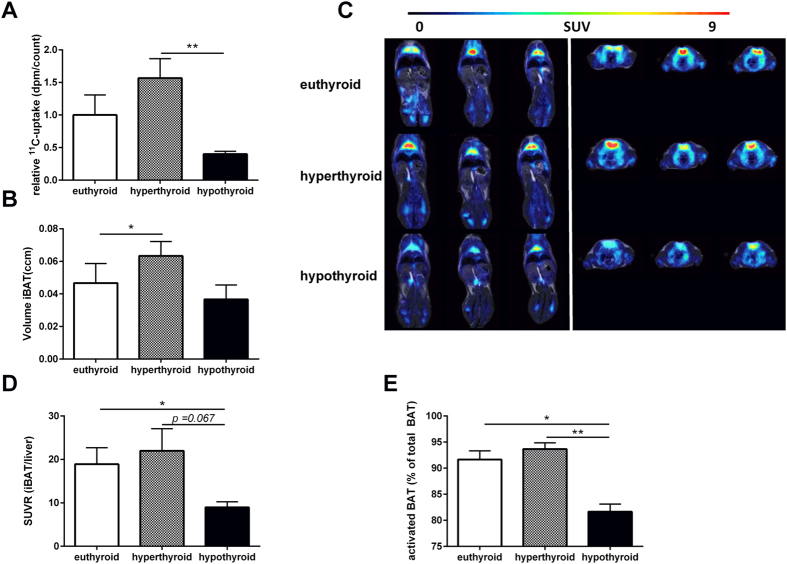
*In-vivo*^18^F-FDG PET/MRI and *ex-vivo*^14^C-acetate uptake assay study for BAT amount and activity. (**A**) *Ex vivo*^14^C-acetate uptake in BAT (n = 10/group). (**B**) Active iBAT volume evaluated by PET/MR imaging. (**C**) Longitudinal (left) and transversal (right) ^18^F-FDG PET/MR fused images and (**D**) SUVR of iBAT in euthyroid, hyperthyroid and hypothyroid mice. (**D**) Proportion of activated BAT (% of total BAT). Data are presented as mean and SEM (*p < 0.05, **p < 0.01, ***p < 0.001).

Given the magnitude of thermogenic gene expression in BAT of hypothyroid mice together with the increased stimulation of the TH-adrenergic axis, hypothyroid mice cannot compensate for hypothermia. Therefore we next addressed whether this is due to defects in BAT activity. First, to determine the oxidative capacity in BAT, we performed *ex-vivo*^14^C-acetate uptake assay and observed a significant increase in fatty acid oxidation in BAT of hyperthyroid mice (74.0 ± 14.0 dpm/count) as compared to hypothyroid mice (18.9 ± 1.95 dpm/count; p < 0.01). There was no significant difference between hypo- and euthyroid mice (47.2 ± 14.5 dpm/count; Fig. 6A). Second, we used a combined PET- and MR-based delineation of active BAT to investigate whether there are differences in BAT formation between the experimental mouse groups. Hyperthyroid mice contained a higher volume of active iBAT (0.06 ccm ± 0.01 ccm) when compared to euthyroid (0.04 ccm ± 0.01 ccm; p < 0.05) and hypothyroid mice (0.03 ccm ± 0.01 ccm; Fig. 6B). We then asked whether the increase in amount of BAT also correlates with increased BAT activity. Metabolically active BAT depots with symmetrical uptake of ^18^F-FDG were observed in all three groups being most marked in the hyperthyroid group (Fig. 6C). The SUVR of ^18^F-FDG accumulation in iBAT was significantly decreased in hypothyroid mice vs. euthyroid mice (8.9 ± 1.3 vs. 18.9 ± 3.8; p < 0.05; Fig. 6D). Furthermore, there was a trend towards an increased ^18^F-FDG accumulation in hyperthyroid compared to euthyroid and hypothyroid mice mice (21.99 ± 5.0 vs. 18.9 ± 3.8 and 8.9 ± 1.3, p = 0.06, respectively).

Finally, to determine to which extent the MR based iBAT volume is metabolically activated, the respective ^18^F-FDG avid iBAT with a SUV threshold of >2 was adjusted to the MR based iBAT volume. Hyperthyroid mice presented with an active iBAT fraction of 94% the highest BAT activity. In contrast, hypothyroid mice showed only in 81% of the interscapular adipose tissue relevant metabolic activity. In iBAT of euthyroid mice we found that 92% is activated (Fig. 6E).

## Discussion

The present study provides new evidence of how TH affects thermogenesis in both brown and white adipose depots. For the first time with regard to metabolic activity of BAT, we were able to identify a distinct profile in hyper- and hypothyroid mice at room temperature. First, regarding the amount of BAT, the *in vivo*^18^F-FDG PET/MRI-estimated BAT volumes gave first evidence for an increased total amount of BAT in hyperthyroid mice compared to hypo- and euthyroid animals (Fig. 6B). Second, although hypo- and euthyroid animals contained detectable BAT, hyperthyroid mice displayed an increased metabolic activity as determined by ^18^F-FDG uptake (Fig. 6C+D). In line with this observation, fatty acid oxidation activity in BAT was increased in hyperthyroid mice as determined by the uptake of ^14^C-acetate (Fig. 6A). The opposite metabolic profile was found in hypothyroid animals, which display significantly lower uptake of ^18^F-FDG and of ^14^C-acetate in BAT when compared to hyperthyroid mice (Fig. 6A–D).

The observation of active BAT in all three experimental groups emphasizes the significance of thermal conditions for metabolic control irrespective of thyroidal state. As discussed by Nedergaard *et al*. under sub-thermal conditions (for mice 18–22 °C) BAT derived heat will be used to maintain body temperature^32^. Paradoxically, the low BAT activity in hypothyroid animals was associated with a strong increase of thermogenic genes (*Ucp1*, *Dio2*, *Fgf21*, *Cidea*, and *Elovl3*; Fig. 5) and with a significant upregulation of the adrenergic system, as evidenced by significant increases of *Ardb3* and NE concentrations in hypothyroid animals (Fig. 4). Although NE *per se* is not an index of NE release or sympathetic tone, these data suggest an increase in norepinephrine outflow to the periphery as a compensatory response to maintain body temperature. This finding is principally in agreement with observations in cold exposed hypothyroid rodents^6^,^33^.

Interestingly, in iWAT and gWAT of hypothyroid mice we detected features of adipose tissue browning, evidenced by an increased expression of brown specific genes (*Ucp1*, *Cidea*, and *Elovl3*; Fig. 2) and a multilocular UCP1-positive phenotype (Fig. 3). Collectively, these data suggest a compensatory WAT browning as a response of decreased heat production due to BAT inactivity in hypothyroid mice. Indeed, decreased BAT thermogenesis in mice lacking bone morphogenetic protein (BMPR1A) is associated with increased circulating NE and a compensatory browning of iWAT and eWAT in a very similar fashion to the hypothyroid mice in the present study^34^. The idea of heat compensation is further substantiated by the observation of enriched expression of the *zinc finger protein 423* (*Zfp423*) in WAT and BAT selectively in hypothyroid mice (Figs 2+5). Gupta *et al*. demonstrated that *Zfp423*+ endothelial cells undergo an endothelial-mesenchymal transition into *Zfp423*+*Pparg*+pericytes that serve as a pool of committed pre-adipocytes^29^. Thus, the increased *Zfp423* expression in the adipose tissues of hypothyroid mice likely suggests the formation of beige adipocytes by recruitment and *de-novo* differentiation of progenitor cells as has been demonstrated by cold exposure or adrenergic *β*_*3*_*-AR* agonist treatment^28^.

The observation of browning of white adipose tissue was also made in white adipose tissues of hyperthyroid mice, where in particular, established markers for adipose tissue browning such as *Ucp1, Fgf21*, *Cidea*, and *Elovl3* were upregulated (Fig. 2). In addition, hyperthyroid mice were characterized by a significant increase in *Ardb1* expression in iWAT (Fig. 3C). Recent studies demonstrated that the absence of the *β*_*1*_*-AR* receptor impairs NE-induced *de-novo* brown adipogenesis in BAT^35^. Conversely, *β*_*1*_*-AR* transgenic mice are resistant to diet-induced obesity and display a high abundance of adipocytes expressing Ucp1 in WAT^36^. With the results gained in the present study we cannot conclude whether or not central effects of T3 contribute to the observed WAT browning. However, evidences from a recent study by Alvarez-Crespo *et al*. suggest a coordinated action of central hyperthyroidism on WAT browning. Thus, the authors for the first time demonstrated that central T3 administration in mice induces UCP1 dependent thermogenesis in BAT and browning of WAT^25^. Also, similar to the observed increased BAT amount in our cohort of hyperthyroid mice, Alvarez-Crespo reported BAT recruitment upon central T3 infusion even at thermoneutrality^25^. Notably, keeping in mind that the hyperthyroid state of our mouse cohort is rather mild it is even more interesting to observe the significant effects on BAT recruitment and WAT browning. Further work will be necessary to address the extent of centrally induced T3 effects, i.e. by the determination of hypothalamic AMPK and ACC in our model of hyperthyroidism^37^.

Collectively, our data provide first evidence for differential mechanisms contributing to WAT browning dependent upon altered thyroidal state. This is most apparent in hypothyroid mice that exhibit increased adipogenesis and *de-novo* differentiation of brown adipocytes potentially as a compensatory mechanism to hypothermia resulting from BAT inactivity. In hyperthyroid mice, it can be hypothesized that increased β-adrenergic activation contributes to WAT browning most likely by central effects of TH. However, it has to be emphasized that with the current data we cannot exclude potential non cell-autonomous contributions from the SNS to adipose tissue browning irrespective of thyroidal state. Thus, in order to rule out that temperature as opposed to thyroidal state is the underlying reason for WAT browning, further studies should be performed under temperatures where sympathetic stimulation of facultative thermogenesis is depressed i.e. thermoneutrality (30 °C)^32^.

## Methods

### Mice

Nine week-old female C57BL/6NTac mice were purchased from Taconic Europe (Lille Skensved, Denmark). The mice were housed in pathogen-free facilities in groups of three at 22 °C with a 12:12-h dark-light cycle (lights on at 06:00 h). Guidelines were approved by the local authorities of the State of Saxony, Germany as recommended by the responsible local animal ethics review board (Regierungspräsidium Leipzig, TVV04/12, Germany). All experiments with mice were carried out according to the approved guidelines.

After one week of adaptation, induction of hyper- or hypothyroidism started at the age of ten weeks with n = 20 mice per experimental group. Mice were rendered hyperthyroid by application of L-thyroxine at a dose of 2 μg/ml diluted in drinking water for 4 weeks^38^. Hypothyroidism was induced by feeding an iodine-free chow diet supplemented with 0.15% propylthiouracil (PTU, catalog TD 97061; Harlan Teklan, Madison, WI, USA) for 4 weeks. Euthyroid mice fed standard chow diet (Altromin GmbH, Lage, Germany) served as controls.

### Body composition

Body weight was recorded once a week and after 4 weeks of treatment. Whole body composition (fat mass, lean mass and total body water) was determined in conscious mice by using nuclear magnetic resonance technology with an EchoMRI700™ instrument (Echo Medical Systems, Houston, TX, USA). Ten animals per experimental group were measured. Rectal body temperature was measured in fed mice between 8:00–9:00 am. Mice were sacrificed by CO_2_ inhalation at the age of 14 weeks.

### ^18^F-FDG PET/MRI of BAT activation

In order to investigate the influence of thyroidal status on BAT activity, small animal PET/magnetic resonance (MR) imaging studies were performed using a dedicated high-resolution scanner (nanoScan, Mediso Medical Imaging Systems, Hungary). Anaesthetized (induction at 4%, maintenance at 1.8% isoflurane in 60%/40% oxygen/air) mice were injected intraperitoneally with 14.5 ± 1.3 MBq ^18^F-FDG followed by a PET/MR scan from 30-60 minute *post-injection* in list mode. The data were reconstructed dynamically into 5-min time frames (OSEM, 4 iterations, 6 subsets, MR-based attenuation and scatter correction).

A T1-weighted MR image (Gradient Echo sequence (GRE), TE = 3.2 ms (out phase), TE = 6.4 ms (in phase), TR = 15.0 ms, Flip Angle = 25°; NEX = 2) served for anatomical orientation with individual PET datasets as well as for threshold based delineation of BAT in the intrascapular region. The ^18^F-FDG uptake was determined using mean standardized uptake values (SUV) in PET/MR based volumes of interest (VOIs) in the intrascapular BAT (iBAT) and liver. To rule out an impact of alterations in the physiological ^18^F-FDG distribution associated with a hyperthyroid or hypothyroid state, ^18^F-FDG uptake was accessed semi-quantitatively. Therefore, SUV ratios (SUVR) were calculated to normalize iBAT uptake to unspecific liver uptake^39^,^40^. Furthermore, iBAT volume was retrieved by a MR-based delineation of fatty tissue in the intrascapular region. Active iBAT volume was defined as iBAT with an increased ^18^F-FDG uptake of SUV > 2. Subsequently, the fraction of active iBAT was calculated as percentage of active iBAT volume out of total adipose tissue volume delineated by MR in the interscapular region. The SUVR and active iBAT volume between the groups were compared using 1-way ANOVA, followed by Bonferroni’s multiple comparison test, using Prism 6.0 (GraphPad, San Diego, Ca, USA). Values of p < 0.05 were considered significant.

### *Ex-vivo*^14^C-acetate loading assay

To quantify the oxidative capacity of WAT and BAT in hyper-, hypo-, and euthyroid mice, we performed an *ex-vivo*^14^C-acetate loading assay that measures the incorporation of radioactively labeled acetate into triacyl glycerides (TAG) as described elsewhere^41^.

### Adipocyte cell size and number

To determine cell size distribution and adipocyte number, 200 μl aliquots of adipocyte suspension were fixed with osmic acid, incubated for 48 h at 37 °C, and counted in a Coulter counter (Multisizer III; Beckman Coulter, Krefeld, Germany)^42^.

### Serum concentrations of thyroxine and serum metabolites

Serum TT_4_ and fT_3_ concentrations were measured using commercial ELISA kits according to the manufacturer’s instructions (DRG Instruments GmbH, Germany). Serum concentrations of epinephrine and norepinephrine were determined using a commercially available ELISA kit (2-CAT (A-N) Research ELISA; Rocky Mountain Diagnostics, Inc., Colorado Springs, USA). The immunoassays were performed according to the manufacturer’s instructions.

### Deiodinase type 2 activity

Activity of type 2 deiodinase were determined in duplicates in BAT homogenates (100 μg protein per 100 μl reaction) as described^43^,^44^.

### Western Blot analysis

Western Blot analysis was performed on samples of inguinal WAT (iWAT), gonadal WAT (gWAT) and BAT as described previously^45^. The following antibodies and dilutions were used: mouse polyclonal anti-Ucp1 antibody (1:500; Merck Millipore; Darmstadt, Germany), pHSL and HSL(1:1000; Cell Signaling Technology, Danvers, USA). Blots were visualized by enhanced chemiluminescence (Pierce, Milwaukee, USA).

### Quantitative real-time-PCR (qPCR)

For quantification of gene expression qPCR was performed using the LightCycler System LC480 and LightCycler-DNA Master SYBR Green I Kit (Roche, Mannheim, Germany) as described previously^46^. Primer sequences are available on request. Gene expression was calculated by the delta-delta CT method using *36B4* as a reference gene^47^. Relative gene expression was calculated by setting the mean of the euthyroid control group to 1 and then calculating each individual value of the three groups of mice studied.

### Immunohistochemistry

Tissues were harvested, fixed in 4% paraformaldehyde (pH 7.4) for 24 h at 4 °C and subsequently embedded in paraffin. Immunohistochemical analysis was performed on BAT and WAT sections rabbit anti-UCP1 polyclonal antibody (ab23841; Abcam, Cambridge, UK). Sections were rinsed thoroughly and incubated with labelled polymer HRP anti-rabbit (Dako Envision™+; Dako, Hamburg, Germany) for 1 h. Visualization was performed with 3,3′-diaminobenzidine. Microscopic examination was performed using an Axio Observer Microscop (Carl Zeiss, Jena, Germany). Images were obtained using ZEN2012 software (Carl Zeiss, Germany).

### Statistical Analyses

Data are shown as mean ± SEM. Statistical significance was determined by Student´s t-test for comparison of two experimental groups or by ANOVA for comparison of multiple conditions followed by Bonferroni’s multiple comparison test, using Prism 6.0 (GraphPad, San Diego, Ca, USA). *P* values less or equal to 0.05 were considered to be significant.

## Additional Information

**How to cite this article**: Weiner, J. *et al*. Thyroid hormone status defines brown adipose tissue activity and browning of white adipose tissues in mice. *Sci. Rep.* **6**, 38124; doi: 10.1038/srep38124 (2016).

**Publisher's note:** Springer Nature remains neutral with regard to jurisdictional claims in published maps and institutional affiliations.
